# Epidemiology and outcomes of marked elevations of alanine aminotransferase >1000 IU/L in an Australian cohort

**DOI:** 10.1002/jgh3.12224

**Published:** 2019-07-18

**Authors:** Danny Con, Andrew Buckle, Amanda J Nicoll, John S Lubel

**Affiliations:** ^1^ Department of Gastroenterology Eastern Health Melbourne Victoria Australia; ^2^ Eastern Health Clinical School Monash University Melbourne Victoria Australia; ^3^ Central Clinical School Monash University Melbourne Victoria Australia

**Keywords:** alanine aminotransferase, alanine transaminase, ischemic hepatitis

## Abstract

**Background and Aim:**

Marked elevations of alanine aminotransferase (ALT) are caused by a limited number of underlying pathologies, including hepatic ischemia, drugs/toxins, viral hepatitis, and—rarely—autoimmune hepatitis. The aim of this study was to determine the relative incidence of pathologies resulting in ALT greater than 1000 IU/L and factors predicting clinical outcomes in an Australian cohort.

**Methods:**

A retrospective cohort study of all adult patients with ALT levels greater than 1000 IU/L between January 2013 and December 2015 was conducted at a large teaching hospital network in Australia. Multivariable logistic regression analysis was used to determine predictors of etiology and mortality.

**Results:**

There were 287 patients identified with ALT levels greater than 1000 IU/L. The most common causes were ischemia (44%), drugs/toxins (19%), biliary obstruction (16%), and viral hepatitis (7%). Independent predictors of a diagnosis of ischemic hepatitis included (adjusted odds ratio; 95% confidence interval): hypotension (29.2; 8.2–104.7), chronic obstructive pulmonary disease (COPD) (20.2; 2.8–145.3), coronary artery disease (12.9; 1.7–98.9), congestive cardiac failure (7.8; 1.2–49.2), diabetes mellitus (7.4; 1.6–33.9), metabolic acidosis (6.2; 2.0–19.4), gamma‐glutamyltransferase < 135 IU/L (5.1; 1.5–17.6), and albumin <34 g/L (3.4; 1.1–11.0). Independent risk factors for all‐cause 28‐day mortality included: septic shock (14.7; 4.3–50.7), metabolic acidosis (7.3; 2.5–21.3), history of COPD (5.4; 1.6–17.8), cardiogenic shock (4.3; 1.6–11.7), prothrombin time ≥ 20 s (3.7; 1.5–9.2), and age ≥ 65 years (3.0; 1.3–7.2).

**Conclusions:**

Ischemic hepatitis was the most common cause of ALT levels greater than 1000 IU/L and was associated with high mortality.

## Graphical abstract

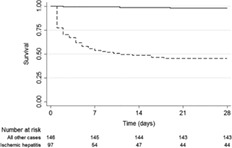

Common causes of marked alanine aminotransferase derangement include liver ischemia, drugs/toxins, biliary obstruction, and viral hepatitis. Determination of etiology and outcomes of patients with severe liver injury can be guided by clinical and biochemical parameters.

## Introduction

Alanine aminotransferase (ALT) is an enzyme found predominantly in the liver and is used as a marker of hepatocellular injury.^1^ Serum ALT concentrations are routinely measured as part of a wider panel of liver function tests in the clinical setting of suspected liver injury.^2^ The upper limit of normal (ULN) ALT has been debated and is dependent on gender, with a ULN of 19 IU/L for women and of 30 IU/L for men.^3^ Reference ranges for ALT may also be dependent on age, ethnicity, and type of assay used.^4^, ^5^ Nonetheless, many laboratories continue to set the ULN serum ALT at around 30–40 IU/L. Mild to moderate elevations of ALT are nonspecific and of limited diagnostic value given the breadth of possible causes.^1^, ^2^ However, marked elevations of ALT over 10 times the ULN have limited etiology and are traditionally thought to be predominantly due to liver ischemia, toxins, or viral hepatitis.^6^

The various causes of marked ALT derangement differ in their prognoses and treatment requirements: ischemic hepatitis is associated with a high mortality and usually requires admission to the intensive care unit (ICU),^7^ whereas viral hepatitis is often managed in the outpatient setting. It is therefore important to establish the diagnosis early in the clinical course in order to predict outcomes and guide management. Previous studies have typically investigated the etiologies of marked liver enzyme derangement over 400–500 IU/L, with few studies having investigated aminotransferase levels of over 1000 IU/L.^8^, ^9^, ^10^, ^11^ Furthermore, to our knowledge, no studies have been performed in an Australian population, and factors predicting clinical outcomes of patients in Australia who develop marked elevations of aminotransferases are unknown.

The aim of this study was therefore to: (i) determine the incidence of ALT greater than 1000 IU/L, (ii) determine the etiology of ALT concentration greater than 1000 IU/L, (iii) describe the clinical and biochemical profiles of the most common etiologies, and (iv) describe the outcomes and factors predicting 28‐day mortality of patients with ALT greater than 1000 IU/L in a large Australian cohort.

## Methods

### *Study design*

We conducted a retrospective cohort study at Eastern Health, a large nontransplant health service that includes three teaching hospitals in Melbourne, Australia, over a 3‐year period between January 2013 and December 2015. The study was approved by the Eastern Health Research Ethics Committee. All cases of serum ALT concentration greater than 1000 IU/L in adult patients aged 18 years and older were retrieved from the hospital pathology database. An ALT cut‐off level of 1000 IU/L has been used in a previous study and is approximately 33 times the ULN.^9^ Electronic medical records were manually reviewed to identify etiologies, comorbidities, clinical signs, biochemical markers, and clinical outcomes.

Relevant comorbid conditions were included if they were recorded in hospital medical notes. Clinical signs including hypotension (systolic blood pressure < 90 mmHg), bradycardia (heart rate < 50 bpm), hypoxia (oxygen saturation < 90%), and fever (temperature ≥ 38.0°C) were included if there was a documented episode occurring within the 48 h preceding the ALT measurement. Biochemical markers including prothrombin time, serum levels of platelets, creatinine, albumin, bicarbonate, total bilirubin, gamma‐glutamyltransferase (GGT), and alkaline phosphatase (ALP) were collected at the time of ALT measurement or at the first available instance within the following 12 h. Survival data were collected with censoring at 28 days from the collection date of ALT greater than 1000 IU/L. Aspartate aminotransferase (AST) data were limited as they are not routinely collected as part of the panel of liver function tests at our health service. We calculated the *R*‐factor as (ALT/ULN of ALT)/(ALP/ULN of ALP). Based on our laboratory's reference range, we used 40 IU/L as the ULN of ALT in males, 30 IU/L as the ULN of ALT in females, and 110 IU/L as the ULN of ALP in all patients.

### *Definitions*

The etiology of marked elevations of ALT was determined according to predetermined criteria^12^, ^13^ (Table S1, Supporting information). Diagnoses were further categorized according to the appropriate clinical setting and clinical impression from medical records, with consensus agreement amongst the authors in the event of ambiguity. Etiology was classified as unknown if more than one diagnosis was likely or if a diagnosis was unable to be made using the predetermined criteria.

### *Statistical analysis*

Normally distributed continuous variables were expressed as mean ± SD and compared using the two‐sided T test. Non‐normally distributed continuous variables were expressed as median with interquartile range and compared using the Mann–Whitney *U* test. Categorical variables were expressed as frequencies and percentages, and comparisons were made using Fisher's exact test. For inclusion in logistic regression analysis, continuous variables were dichotomized using clinically relevant categories and/or receiver operating characteristic analysis^14^, ^15^ where appropriate. Univariable logistic regression was used to identify factors associated with a diagnosis of ischemic hepatitis and all‐cause 28‐day mortality. All factors with *P* < 0.1 were included in multivariable logistic regression models that were adjusted for age ≥ 65 years and gender as prespecified covariates. Two models were used: (i) a full model, and (ii) a reduced model using backward stepwise selection with removal criterion *P* > 0.05. Kaplan–Meier analysis using the log‐rank test was used to estimate and compare survival curves with a serial time of 28 days. A two‐sided *P* < 0.05 was used to indicate statistical significance. Statistical analysis was performed using Stata/IC 14.1 (StataCorp LLC, 2016 College Station, TX, USA).

## Results

### *Incidence and demographics*

There were 287 patients with an ALT level greater than 1000 IU/L identified amongst 388 267 acute admissions to our health service between January 2013 and December 2015. The incidence of ALT levels over 1000 IU/L was therefore 7.4 cases per 10 000 admissions per year. Baseline demographic data were obtained (Table 1). In 127 patients (44.3%), there were no comorbidities; 73 patients (25.4%) had one comorbidity, and 87 patients (30.3%) had two or more comorbidities.

**Table 1:** Baseline demographic data characteristics of patients with serum ALT >1000 IU/L

Demographics	*n* (%)
Age (years), mean ± SD	56.8 ± 21.1
Females	160 (55.8)
Past or current smoker	87 (30.3)
History of coronary artery disease	39 (13.6)
History of CCF	52 (18.1)
History of COPD	40 (13.9)
History of CKD	28 (9.8)
History of CLD	28 (9.8)
History of cerebrovascular disease	19 (6.6)
History of diabetes mellitus	52 (18.1)
History of cancer	42 (14.6)
History of chronic viral hepatitis	21 (7.3)
Total	287 (100.0)

ALT, alanine aminotransferase; CCF, congestive cardiac failure; CKD, chronic kidney disease; CLD, chronic liver disease; COPD, chronic obstructive pulmonary disease.

### *Etiology*

**Figure 1:**
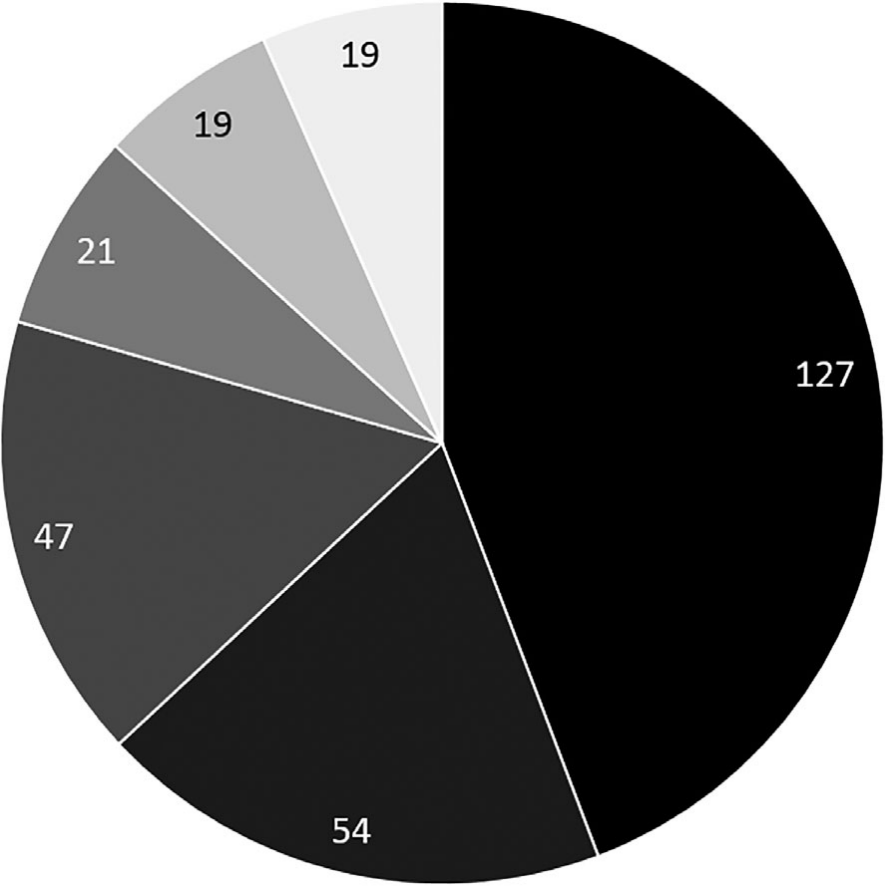
Etiology of serum ALT level > 1000 IU/L. (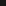), Ischemia (44%); (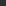), drugs/toxins (19%); (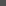), biliary obstruction (16%); (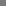), viral (7%); (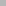), other (7%); (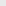), unclear diagnosis (7%).

Of the 287 cases, etiology was determined in 268, while no clear diagnosis was made in the remaining 19 (Fig. 1). The four most common causes were acute ischemia (127; 44.3%), drugs/toxins (54; 18.8%), extrahepatic biliary obstruction (47; 16.4%), and viral hepatitis (21; 7.3%).

#### *Ischemic hepatitis*

Of the 127 cases of acute ischemia, 65 were due to cardiogenic shock (25 acute myocardial infarction, 22 cardiac arrest, 7 exacerbation of congestive cardiac failure [CCF], 5 arrhythmia, 3 pulmonary embolism, 2 cardiac tamponade, 1 Takotsubo cardiomyopathy), 49 were due to septic shock (27 pneumonia, 10 intra‐abdominal sepsis, 5 urosepsis, 7 other sources), 12 were due to hypovolemic shock (9 hemorrhagic, 3 gastrointestinal losses), and 1 was due to anaphylactic shock.

#### *Toxic hepatitis*

Of the 54 cases of toxic hepatitis, 28 were due to paracetamol toxicity, 7 were attributed to antibiotics (3 amoxicillin‐clavulanate, 1 piperacillin‐tazobactam, 1 erythromycin, 1 minocycline, 1 rifampicin), 3 were due to 3,4‐methylenedioxymethamphetamine (Ecstasy), 1 was due to alcohol, and 15 were due to others (2 amiodarone, 2 herbal teas, 1 methadone, 1 sulfasalazine, 1 rosuvastatin, 1 carbamazepine, 1 duloxetine, 1 amitriptyline, 1 methotrexate, 4 unknown drug due to multiple coingestion).

#### *Biliary obstruction*

Of the 47 obstructive causes, 33 were due to choledocholithiasis (without cholangitis), 8 were due to ascending cholangitis, 4 were due to a pancreatic mass, 1 was due to cholangiocarcinoma, and 1 was due to biliary stricture.

#### *Viral hepatitis*

Of the 21 viral causes, 8 were due to hepatitis C, 6 were due to hepatitis B, 3 were due to hepatitis A, 2 were due to cytomegalovirus, and 2 were due to Epstein–Barr virus.

#### *Other causes*

Of the remaining 19 identifiable causes, 6 were due to direct liver insult (4 hepatectomy, 2 liver hemorrhage), 5 were due to rhabdomyolysis, 3 were due to autoimmune conditions (2 autoimmune hepatitis, 1 primary sclerosing cholangitis), 3 were due to pregnancy‐related conditions (2 cholestasis of pregnancy, 1 pre‐eclampsia), 1 was due to Wilson's disease, and 1 was due to leptospirosis.

### *Clinical and biochemical profile by etiology*

Patients with deranged ALT due to ischemic hepatitis were older (mean age 67.3 years, SD 18.2) compared to patients with all other causes (mean age 48.4 years, SD 19.6; *P* < 0.001) and were more likely to have had a documented episode of hypotension, bradycardia, and hypoxia (Table 2). There were no significant differences in the prevalence of smoking, chronic viral hepatitis, and chronic liver disease across etiologies; however, patients with ischemic hepatitis were more likely to have CCF, chronic obstructive pulmonary disease (COPD), coronary artery disease, diabetes mellitus, cerebrovascular disease, chronic kidney disease (CKD), and cancer.

**Table 2:** Comparison of clinical characteristics of patients with alanine aminotransferase >1000 IU/L due to ischemic hepatitis compared to all other causes

Clinical characteristic	Ischemic hepatitis	All other causes	*P*
Hypotension, *n* (%)	98 (77.2)	14 (8.8)	<0.001
Bradycardia, *n* (%)	27 (21.3)	9 (5.6)	<0.001
Hypoxia, *n* (%)	55 (43.3)	2 (1.3)	<0.001
Fever, *n* (%)	25 (19.7)	22 (13.8)	0.20

The biochemical profiles of the four most common conditions were elicited (Table 3). Serum ALT was lower in patients with biliary obstruction, while there was no difference between patients with ischemic, toxic, or viral hepatitis. Patients with ischemic hepatitis had lower ALP compared to obstructive and viral hepatitis but not toxic hepatitis. Patients with ischemic hepatitis had lower bicarbonate, albumin, bilirubin, and GGT but higher creatinine and prothrombin time compared to other causes. Platelet count was lower in ischemic hepatitis than toxic and obstructive hepatitis but not viral hepatitis. Patients with viral hepatitis had lower AST compared to other causes.

**Table 3:** Biochemical values by etiology of ALT >1000 IU/L with comparisons made against ischemic hepatitis

Biochemistry	Ischemic	Toxic	Obstructive	Viral
Platelets, ×10^9^/L				
Mean ± SD	164.1 ± 8.3	201.5 ± 11.7	244.2 ± 11.8	184.5 ± 12.6
*P* value	—	0.012	<0.001	0.34
Bicarbonate, mmol/L				
Mean ± SD	18.9 ± 0.6	23.3 ± 0.7	25.0 ± 0.4	27.0 ± 0.7
*P* value	—	0.001	<0.001	<0.001
Creatinine, μmol/L				
Mean ± SD	178.7 ± 8.1	98.2 ± 11.2	73.4 ± 2.7	80.4 ± 3.9
*P* value	—	<0.001	<0.001	<0.001
Albumin, g/L				
Mean ± SD	28.7 ± 0.6	35.8 ± 0.7	38.1 ± 0.6	36.1 ± 1.0
*P* value	—	<0.001	<0.001	<0.001
Prothrombin time, s				
Median (IQR)	23.2 (18.9–31.0)	19.6 (14.8–24.9)	13.3 (12.8–15.0)	14.0 (13.4–16.3)
*P* value	—	0.005	<0.001	<0.001
Bilirubin, μmol/L				
Median (IQR)	21 (10–37)	28 (14–64)	43 (29–87)	76 (36–155)
*P* value	—	0.048	<0.001	<0.001
ALT, IU/L				
Median (IQR)	1451 (1166–2340)	1591 (1162–3425)	1199 (1055–1471)	1790 (1244–2472)
*P* value	—	0.19	<0.001	0.23
AST, IU/L (*n* = 64)				
Median (IQR)	2442 (1509–3448)	2086, 723–4731	2751, 822–3009	1148, 983–1580
*P* value	—	0.49	0.76	0.012
ALP, IU/L				
Median (IQR)	117 (76–199)	124 (83–199)	221 (166–316)	189 (157–233)
*P* value	—	0.91	<0.001	0.003
GGT, IU/L				
Median (IQR)	108 (53–246)	171 (79–375)	581 (303–1068)	245 (202–426)
*P* value	—	0.009	<0.001	<0.001
R‐factor				
Median (IQR)	44.4 (22.8–81.4)	48.6 (29.8–99.7)	18.7 (13.8–27.7)	29.1 (21.2–38.0)
*P* value	—	0.13	<0.001	0.033

ALP, alkaline phosphatase; ALT, alanine aminotransferase; AST, Aspartate aminotransferase; GGT, gamma‐glutamyltransferase; IQR, interquartile range.

### *Predictors of etiology*

In the full multivariable logistic regression model, medical comorbidities that constituted independent risk factors for ischemic hepatitis included history of COPD, CCF, and diabetes mellitus; independently associated clinical factors included hypotension and hypoxia, while independently associated biochemical abnormalities included metabolic acidosis and lower GGT (Table 4). All significant variables in the full model, apart from hypoxia, remained in the reduced model. Coronary artery disease and albumin <34 g/L did not reach significance in the full model but remained in the reduced model as significant independent predictors.

**Table 4:** Factors associated with a diagnosis of ischemic hepatitis from univariable and multivariable logistic regression analysis

Characteristic	Univariate analysis			Full multivariable model†			Reduced multivariable model‡		
	OR	95% CI	*P*	Adjusted OR	95% CI	*P*	Adjusted OR	95% CI	*P*
Age ≥ 65 years	7.6	4.5–13.1	<0.001	2.7	0.6–12.3	0.21	—	—	—
Male	1.2	0.8–2.0	0.36	1.3	0.4–4.7	0.70	—	—	—
COPD	11.8	4.5–31.2	<0.001	20.2	2.0–198.6	0.010	20.2	2.8–145.3	0.003
CCF	12.0	5.2–27.8	<0.001	12.1	1.5–99.8	0.021	7.8	1.2–49.2	0.028
Coronary artery disease	5.2	2.4–11.4	<0.001	7.8	0.7–82.7	0.09	12.9	1.7–98.9	0.014
Cerebrovascular disease	7.5	2.1–26.5	0.002	3.6	0.1–108.3	0.46	—	—	—
CKD	6.9	2.5–18.6	<0.001	0.2	0.01–1.7	0.13	—	—	—
Diabetes mellitus	3.6	1.9–6.8	<0.001	8.8	1.7–45.9	0.010	7.4	1.6–33.9	0.010
Cancer	2.3	1.1–4.7	0.021	0.6	0.1–3.4	0.58	—	—	—
Hypotension	35.2	17.7–70.1	<0.001	53.2	10.4–272.1	<0.001	29.2	8.2–104.7	<0.001
Hypoxia	60.3	14.3–254.2	<0.001	23.2	1.9–288.0	0.015	—	—	—
Bradycardia	4.5	2.0–10.0	<0.001	0.5	0.04–5.7	0.54	—	—	—
Platelets <180 × 10^9^/L	2.8	1.7–4.5	<0.001	0.6	0.2–2.1	0.40	—	—	—
PT ≥ 20 s	5.6	3.1–9.9	<0.001	2.6	0.6–12.4	0.22	—	—	—
Albumin <34 g/L	10.8	6.2–18.8	<0.001	3.6	0.9–14.3	0.065	3.4	1.1–11.0	0.039
Creatinine ≥110 μmol/L	17.9	9.9–32.8	<0.001	1.0	0.3–3.9	0.96	—	—	—
Bicarbonate <22 mmol/L	9.7	5.5–17.0	<0.001	6.8	1.6–28.9	0.010	6.2	2.0–19.4	0.002
GGT < 135 IU/L	5.3	3.2–8.8	<0.001	4.5	1.0–19.2	0.044	5.1	1.5–17.6	0.010
Bilirubin <22 μmol/L	3.1	1.9–5.1	<0.001	3.9	0.9–17.5	0.075	—	—	—

† All variables with *P* < 0.1 on univariate analysis, as well as prespecified additional variables of age ≥65 years and gender, were entered into a full multivariable logistic regression model.

‡ The full multivariable model was entered into a backward stepwise logistic regression model with removal criterion *P* > 0.05, leaving a reduced model with all remaining variables with *P* < 0.05.

CCF, congestive cardiac failure; CI, confidence interval; CKD, chronic kidney disease; COPD, chronic obstructive pulmonary disease; GGT, gamma‐glutamyltransferase; OR, odds ratio; PT, prothrombin time.

### *Clinical outcomes*

The mean length of hospital stay was 6.9 days (SD 6.9), and there was no significant difference in length of stay across etiologies. Eighty‐six (30.0%) patients were admitted to ICU, and the mean length of ICU stay was 3.5 days (SD 3.6). Patients with ischemic hepatitis were more likely to be admitted to ICU compared to patients with toxic hepatitis (54.3 *vs* 14.8%; odds ratio [OR] 6.8, 95% confidence interval [CI] 3.0–15.7, *P* < 0.001) and biliary obstruction (4.3%; OR 26.8, 95% CI 6.2–115.1, *P* < 0.001). There was no difference in length of ICU stay across etiologies. No patients received a liver transplant in the study period.

**Figure 2:**
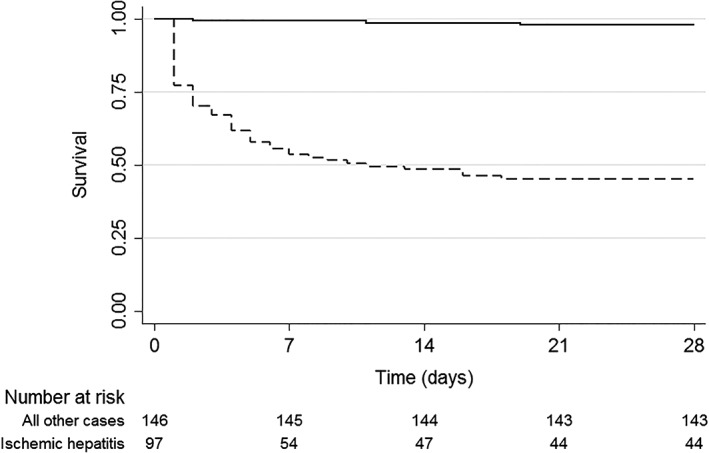
Kaplan–Meier survival estimates for ischemic hepatitis *versus* all other causes of ALT concentration > 1000 IU/L (*p* < 0.001 by the log rank test). (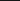) All other causes; (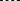), ischemic hepatitis.

All‐cause 28‐day mortality data were available for 269 cases (93.4%). The overall 28‐day mortality rate was 30.5% (82 of 269), and almost all cases were patients with ischemic hepatitis (78 of 82; 95.1%). The remaining deaths were caused by toxic hepatitis in two cases, obstructive hepatitis in one case, and viral hepatitis in one case. The most common causes of death were cardiogenic shock (43.9%), septic shock (41.5%), and hemorrhagic shock (6.1%). Patients with ischemic hepatitis had a higher mortality compared to all other causes (*P* < 0.001), with a median survival of 11 days (95% CI 5–17) (Fig. 2).

### *Predictors of mortality*

A full comparison of clinical characteristics between patients with markedly deranged ALT who died within 28 days from any cause and those who survived can be found in Table S2. In the full multivariable logistic regression model (Table 5), significant predictors of all‐cause 28‐day mortality included: age ≥ 65 years, septic shock, cardiogenic shock, history of COPD, history of diabetes mellitus, elevated prothrombin time ≥ 20 s, and metabolic acidosis. Renal failure approached but did not reach significance at the 95% confidence level, with a point estimate of the OR of 2.9 (95% CI 0.9–9.2). All variables apart from diabetes mellitus that were significant predictors in the full model remained in the reduced model.

**Table 5:** Odds ratios (OR) and adjusted OR of 28‐day mortality in patients with ischemic hepatitis from univariate and multivariable logistic regression

	Univariate analysis			Full multivariable model†			Reduced multivariable model‡		
Characteristic	OR	95% CI	*P*	Adjusted OR	95% CI	*P*	Adj. OR	95% CI	*P*
Age ≥ 65 years	6.4	3.6–11.4	<0.001	3.5	1.1–10.6	0.030	3.0	1.3–7.2	0.014
Male	1.5	0.9–2.5	0.13	2.4	0.8–7.1	0.13	—	—	—
Septic shock	12.0	5.8–24.9	<0.001	13.0	3.0–55.9	0.001	14.7	4.3–50.7	<0.001
Cardiogenic shock	2.6	1.3–5.0	0.005	3.9	1.1–13.7	0.035	4.3	1.6–11.7	0.005
History of COPD	4.7	2.3–9.7	<0.001	8.6	2.0–36.8	0.004	5.4	1.6–17.8	0.006
History of CCF	7.1	3.6–13.8	<0.001	2.6	0.8–9.2	0.13	—	—	—
History of CAD	3.9	1.9–8.0	<0.001	0.8	0.2–3.8	0.76	—	—	—
History of diabetes mellitus	2.0	1.1–3.7	0.031	0.3	0.1–1.0	0.044	—	—	—
History of CKD	3.9	1.7–8.8	0.001	0.3	0.1–1.3	0.11	—	—	—
History of CVD	3.5	1.3–9.0	0.010	2.3	0.4–12.1	0.33	—	—	—
Platelets < 180 × 10^9^/L	2.1	1.2–3.6	0.006	0.7	0.2–2.2	0.57	—	—	—
Creatinine ≥ 110 μmol/L	16.6	8.5–32.5	<0.001	2.9	0.9–9.2	0.070	—	—	—
Prothrombin time ≥ 20 s	8.4	4.3–16.4	<0.001	3.5	1.0–11.7	0.042	3.7	1.5–9.2	0.006
Albumin < 34 g/L	6.6	3.5–12.3	<0.001	1.2	0.4–4.0	0.74	—	—	—
GGT < 135 IU/L	2.8	1.7–4.9	<0.001	0.5	0.2–1.6	0.26	—	—	—
Bicarbonate < 22 mmol/L	10.1	5.5–18.6	<0.001	9.1	2.5–32.5	0.001	7.3	2.5–21.3	<0.001
Bilirubin < 22 μmol/L	1.6	1.0–2.7	0.073	1.0	0.3–3.2	0.98	—	—	—

† All variables with *P* < 0.1 on univariate analysis, as well as prespecified additional variables of age ≥65 years and gender, were entered into a full multivariable logistic regression model.

‡ The full multivariable model was entered into a backward stepwise logistic regression model with removal criterion *P* > 0.05, leaving a reduced model with all remaining variables with *P* < 0.05.

CAD, coronary artery disease; CCF, congestive cardiac failure; CI, confidence interval; CKD, chronic kidney disease; COPD, chronic obstructive pulmonary disease; CVD, cerebrovascular disease; GGT, gamma‐glutamyltransferase.

## Discussion

This study is the first to elicit the etiology and clinical outcomes of patients with marked elevations of serum ALT in an Australian population. The most common cause of ALT greater than 1000 IU/L was ischemic hepatitis, which was associated with a high mortality. This is consistent with previous studies in other regions of the world, where ischemic hepatitis accounts for 15–74% of patients with marked elevations of aminotransferases.^8^, ^9^, ^10^, ^11^, ^16^, ^17^, ^18^ However, varying concentrations were used to indicate aminotransferase derangement, and the relative frequency of ischemic hepatitis appears to increase with greater ALT and AST elevation.^7^ Drugs and toxins were found to be the second most common cause of marked ALT derangement in our study, which is consistent with a previous study in Ireland.^9^ On the other hand, viral hepatitis was a relatively uncommon cause in our population, which may be explained by comparatively lower rates of chronic infection in Western countries.^19^

Biliary obstruction is not traditionally considered to be a common cause of marked aminotransferase derangement^6^ but was the third most common cause in this study. Marked elevations of liver enzymes is an uncommon but increasingly recognized manifestation of choledocholithiasis, where up to 6% of patients develop ALT or AST over 1000 IU/L.^20^, ^21^, ^22^ Concentrations of aminotransferases above 400–500 IU/L are caused by biliary obstruction in 20–34% of cases in Western countries^11^, ^17^, ^23^ and in 12% of cases in China.^24^ However, biliary obstruction is rare at higher concentrations of aminotransferases, where choledocholithiasis causes only 4% of ALT greater than 1000 IU/L in Ireland^9^ and only 1% of ALT or AST over 3000 IU/L in Singapore.^8^ It is therefore surprising that, in this study, serum ALT greater than 1000 IU/L was caused by biliary obstruction in 16% of cases. The relative frequency of choledocholithiasis and cholangitis in our population may suggest a higher prevalence of gall stone disease. Extrahepatic biliary obstruction should therefore be high on the list of differential diagnoses considered by Australian clinicians even at ALT concentrations >1000 IU/L.

Our study also demonstrates that each etiology has a distinct biochemical profile that may aid in diagnosis. Patients with ischemic hepatitis were more likely to have metabolic acidosis, renal failure, and low albumin, which are suggestive of a more severe pathology. Prothrombin time was elevated in most patients with ischemic and toxic hepatitis, but the degree of derangement was greater in ischemic hepatitis. Patients with ischemic hepatitis were more likely to have a normal bilirubin concentration, where the median bilirubin in patients with obstructive and viral hepatitis was more than two and three times greater, respectively. Serum ALT was lower in patients with biliary obstruction; however, the utility of ALT in establishing a diagnosis is questionable given the large spread of ALT concentrations across pathologies. Although platelet count was lower in patients with ischemic hepatitis, this is unlikely to be useful as platelet count was mostly within the normal limits in all pathologies. Furthermore, although the *R*‐factor was lower in patients with an obstructive cause, the majority of patients regardless of etiology had *R*‐factors exceeding the accepted cut‐off of 5 to indicate a hepatocellular pattern of liver injury. This is expected given that our cohort was selected specifically to include patients with markedly elevated ALT. Hence, the *R*‐factor is unlikely to be useful in distinguishing the etiology of marked ALT derangement.

This study is the first to explore independent predictors of etiology in marked liver enzyme derangement in an Australian cohort. Clinical signs that predict a diagnosis of ischemic hepatitis were hypotension and hypoxia, which are unsurprising. However, in more than one in five cases, a preceding episode of hypotension was undetected, and thus, clinicians should not discount the possibility of an ischemic event simply because of the absence of a documented hypotensive episode. A diagnosis of ischemic hepatitis is independently predicted by a history of COPD, CCF, and diabetes mellitus, which were the three most common comorbidities in patients with liver ischemia, and may all contribute to hemodynamic failure. Of the biochemical markers, metabolic acidosis and GGT under 135 IU/L were predictive of ischemic hepatitis, where GGT concentration had a higher degree of derangement in other pathologies. Renal failure conferred significant odds of having ischemic hepatitis on univariable analysis but was not an independent predictor in this study. This may be explained by a lack of specification of the acuity of renal failure in our study, although we did adjust for the presence of CKD.

Marked ALT derangement was associated with a high mortality in this study and was strongly dependent on etiology. Independent predictors of 28‐day mortality included septic shock, cardiogenic shock, history of COPD, metabolic acidosis, raised prothrombin time, and older age. Previous studies in patients with ischemic hepatitis have identified various independent risk factors of mortality: older age, higher ICU severity scores, septic shock, higher international normalized ratio, renal failure, higher bilirubin, higher lactate dehydrogenase, lower albumin, and requirement of vasopressors.^8^, ^25^, ^26^, ^27^, ^28^ However, the study populations have been heterogeneous and typically included ICU patients or patients with severe liver injury with ALT over 3000–4000 IU/L, and none have been conducted on an Australian population. Our findings have important clinical relevance and suggest that marked ALT derangement warrants urgent investigation to elicit etiology as they confer vastly different mortality risks.

### *Limitations*

The study was conducted at a single health service that may limit the external validity of the results. However, we expect that our results may be generalizable to suburban metropolitan areas of major cities in Australia given similar demographic distributions across the country. The study was retrospective and therefore harbors limitations inherent to all retrospective study designs. In particular, information (misclassification) bias may be present, in part because clinical interpretation of previous events through chart reviews may be subjective. However, this was mitigated through the use of our predefined diagnostic criteria for the etiology of marked ALT derangement. Certainly, the independent variables that emerged from our analysis cannot be considered causative.

### *Conclusions*

Marked elevation in ALT reflects severe liver injury and has a high mortality rate. Common causes of marked ALT derangement include liver ischemia, drugs/toxins, biliary obstruction, and viral hepatitis. The relative frequencies of etiology likely reflect the regional incidences of each condition. Ischemic hepatitis is associated with a high mortality and accounts for the vast majority of deaths following marked ALT derangement. A diagnosis of ischemic hepatitis can be predicted by important clinical information and has a distinct biochemical profile. Biliary obstruction is an underrecognized but common cause of marked ALT derangement in an Australian population. Early diagnosis and identification of high‐risk patients are required to mitigate the high mortality associated with severe liver injury.

## Supporting information


**Table S1.** Criteria for retrospective diagnosis of etiology of serum alanine aminotransferase (ALT) concentration > 1000 IU/L.
**Table S2.** Comparison of clinical characteristics of patients with alanine aminotransferase >1000 IU/L who died within 28 days of any cause compared with those who survived.
