# Supplementary material for: Epidemiology and outcomes of marked elevations of alanine aminotransferase >1000 IU/L in an Australian cohort
Source: JGH Open. 2019 Jul 18;4(2):106–12. doi: 10.1002/jgh3.12224 (PMC7144769; doi:10.1002/jgh3.12224)
Supplement: Supplementary file 1 — Table S1. Criteria for retrospective diagnosis of etiology of serum alanine aminotransferase (ALT) concentration > 1000 IU/L. Table S2. Comparison of clinical characteristics of patients with alanine aminotransferase >1000 IU/L who died within 28 days of any cause compared with those who survived. [file JGH3-4-106-s001.docx]

Supporting Information Table 1. Criteria for retrospective diagnosis of etiology of serum ALT concentration >1,000 IU/L.

| **Diagnosis** | **Criteria** |
| --- | --- |
| Ischemic hepatitis | Appropriate clinical setting of cardiac, respiratory or circulatory failure; sudden and transient rise in ALT; clinical impression from medical records, and exclusion of other causes of acute hepatitis. |
| Toxic hepatitis | Roussel Uclaf Causality Assessment Method (RUCAM) score for drug induced liver injury greater than 8 (highly probable); or 6–8 (probable) in addition to consensus impression from the medical records and study authors. |
| Viral hepatitis | Elevated immunoglobulin M levels specific to one of hepatitis A virus (HAV), cytomegalovirus (CMV) or Epstein-Barr virus (EBV), or positive hepatitis B surface antigen (HBsAg), or positive hepatitis C virus (HCV) RNA polymerase chase reaction (PCR), or positive hepatic E virus (HEV) RNA PCR; clinical impression from medical records, and; exclusion of other causes of acute hepatitis. |
| Rhabdomyolysis | Elevated creatine kinase concentration >1,000 IU/L in the absence of concurrent myocardial infarction; appropriate clinical setting, and; clinical impression from medical records. |
| Extrahepatic biliary obstruction | Radiology (computed tomography, ultrasound or magnetic resonance imaging) proven common bile duct obstruction or dilated common bile duct diameter >10mm, and; exclusion of other causes of acute hepatitis. |
| Other conditions | Appropriate clinical setting; clinical impression from medical records, and; exclusion of other causes of acute hepatitis. |

Supporting Information Table 2. Comparison of clinical characteristics of patients with ALT >1,000 IU/L who died within 28 days of any cause compared with those who survived.

| **Characteristic** | **Died within 28 days** | **Survived** | ***p*** |
| --- | --- | --- | --- |
| Age, years (mean ± SD) | 70.6 ± 16.9 | 51.1 ± 19.9 | <0.001 |
| Females, *n* (%) | 41 (50.0) | 112 (59.9) | 0.14 |
| Past or active smoker, *n* (%) | 25 (30.5) | 57 (30.5) | >0.99 |
| History of COPD, *n* (%) | 24 (29.3) | 15 (8.0) | <0.001 |
| History of CCF, *n* (%) | 34 (41.5) | 17 (9.1) | <0.001 |
| History of coronary artery disease, *n* (%) | 22 (26.8) | 16 (8.6) | <0.001 |
| History of cerebrovascular disease, *n* (%) | 11 (13.4) | 8 (4.3) | 0.017 |
| History of CLD, *n* (%) | 10 (12.2) | 17 (9.1) | 0.51 |
| History of CKD, *n* (%) | 16 (19.5) | 11 (5.9) | 0.001 |
| History of diabetes mellitus, *n* (%) | 22 (26.8) | 29 (15.5) | 0.042 |
| History of cancer, *n* (%) | 13 (15.9) | 27 (14.4) | 0.85 |
| History of chronic viral hepatitis, *n* (%) | 4 (4.9) | 17 (9.1) | 0.33 |
| Cardiogenic shock, n (%) | 36 (43.9) | 27 (14.4) | <0.001 |
| Septic shock, n (%) | 34 (41.5) | 12 (6.4) | <0.001 |
| Hypovolemic shock, n (%) | 5 (6.1) | 4 (2.1) | 0.14 |
| Platelets, ×10^9^/L (mean ± SD) | 162.0 ± 88.2 | 201.2 ± 82.9 | <0.001 |
| Bicarbonate, mmol/L (mean ± SD) | 17.7 ± 7.6 | 23.8 ± 4.7 | <0.001 |
| Creatinine, µmol/L (mean ± SD) | 191.8 ± 89.9 | 101.1 ± 71.5 | <0.001 |
| Albumin, g/L (mean ± SD) | 28.3 ± 6.5 | 35.0 ± 5.7 | <0.001 |
| Prothrombin time, s (median, IQR) | 26.2 (20.7–35.0) | 15.9 (13.8–22.3) | <0.001 |
| Bilirubin, µmol/L (median, IQR) | 25 (14–39) | 31 (14–55) | 0.13 |
| ALT, IU/L (median, IQR) | 1457 (1165–2539) | 1386 (1132–2028) | 0.31 |
| ALP, IU/L (median, IQR) | 154 (94–218) | 150 (98–233) | 0.53 |
| GGT, IU/L (median, IQR) | 95 (56–260) | 235 (108–501) | <0.001 |
| Length of hospital admission, days (mean ± SD) | 4.5 (6.1) | 7.9 (7.0) | <0.001 |
| Admission to ICU, *n* (%) | 46 (56.1) | 37 (19.8) | <0.001 |
| Length of ICU stay, days (mean ± SD) | 2.9 ± 2.8 | 4.5 ± 4.3 | 0.053 |
| Total, *n* (%) | 82 | 187 | - |
